# Anti-glomerular basement membrane nephritis following IgA vasculitis in a child: a diagnostic challenge

**DOI:** 10.1007/s00467-026-07337-z

**Published:** 2026-05-05

**Authors:** Rika Tanaka, Akinori Miyazono, Takuro Mitsunobu, Noriko Uesugi, Yasuhiro Okamoto

**Affiliations:** 1 Department of Pediatrics, Kagoshima University Hospital, 8-35-1 Sakuragaoka, Kagoshima, 890-8520 Japan; 2 Department of Pathology, Faculty of Medicine, Fukuoka University, Fukuoka, Japan

**Keywords:** Anti-glomerular basement membrane disease, IgA vasculitis, Rapidly progressive glomerulonephritis, Early diagnosis, COVID-19

## Abstract

Anti-glomerular basement membrane (anti-GBM) nephritis is a rare but highly aggressive cause of rapidly progressive glomerulonephritis (RPGN). Early recognition is essential, as delayed diagnosis leads to irreversible kidney damage and poor prognosis. We report an 8-year-old girl with anti-GBM nephritis initially misattributed to kidney involvement of IgA vasculitis (IgAV). Diagnosis was confirmed by markedly elevated anti-GBM antibodies and kidney biopsy findings. Despite plasma exchange and immunosuppressive therapy, kidney function deteriorated to kidney failure within several months. This case provides an important clinical insight for pediatric nephrologists: worsening urinary findings or kidney function during follow-up of IgAV should prompt consideration of RPGN and appropriate serological screening, as well as timely kidney biopsy, rather than being attributed solely to IgAV nephritis.

## Introduction

Anti-glomerular basement membrane (anti-GBM) nephritis is a rare autoimmune disease, particularly in children [1]. It is one of the most aggressive forms of glomerulonephritis, typically presenting as rapidly progressive glomerulonephritis (RPGN) and occasionally complicated by life-threatening alveolar hemorrhage [1]. Kidney prognosis is largely determined by the extent of irreversible glomerular damage at diagnosis, particularly crescent formation and global glomerulosclerosis. Reflecting this, the Kidney Disease: Improving Global Outcomes (KDIGO) guidelines recommend against aggressive therapy in patients requiring dialysis at presentation and show extensive chronic lesions in the absence of alveolar hemorrhage [2]. These recommendations highlight the critical importance of establishing the diagnosis before advanced and irreversible kidney injury develops. Here, we report a pediatric case of anti-GBM nephritis that developed during follow-up for kidney involvement associated with IgA vasculitis (IgAV), in which diagnostic delay contributed to advanced kidney damage.

## Case report

An 8-year-old girl presented with ankle arthritis and palpable purpura on her lower extremities, without thrombocytopenia, and was clinically diagnosed with IgA vasculitis (IgAV) at the referring hospital. Initial serum creatinine (Cr) level was 0.36 mg/dL, and urinalysis showed hematuria (3 +) and trace proteinuria, corresponding to red blood cells (RBC) 30–39/high-power field (HPF) and urinary protein-to-creatinine ratio (UPCR) of 0.26 g/g･Cr. Urinalysis was performed every 1–2 weeks to monitor for IgAV nephritis (IgAVN). Findings remained stable with RBC 5–9/HPF to 30–49/HPF and UPCR around 0.30 g/g･Cr. Three months later, she developed coronavirus disease 2019 (COVID-19). Fifteen days after COVID-19 onset, urinary findings worsened to RBC 50–99/HPF and UPCR 1.15 g/g･Cr, and serum Cr increased to 0.74 mg/dL, considered a flare of IgAVN. Thirty-six days after COVID-19 onset, urinalysis worsened (RBC 50–99/HPF, UPCR 3.92 g/g･Cr), and kidney function rapidly deteriorated (BUN 54 mg/dL, Cr 3.23 mg/dL). Despite intravenous fluid therapy, kidney function did not improve, and she was transferred to our institution.

A comprehensive workup for RPGN was promptly initiated, including measurements of anti-double-stranded DNA antibodies, myeloperoxidase-ANCA, proteinase 3-ANCA, complement levels, and anti-GBM antibodies. All results, except for anti-GBM antibodies, were within normal ranges. The anti-GBM antibody test was outsourced to an external laboratory, and the result was pending.

**Fig. 1:**
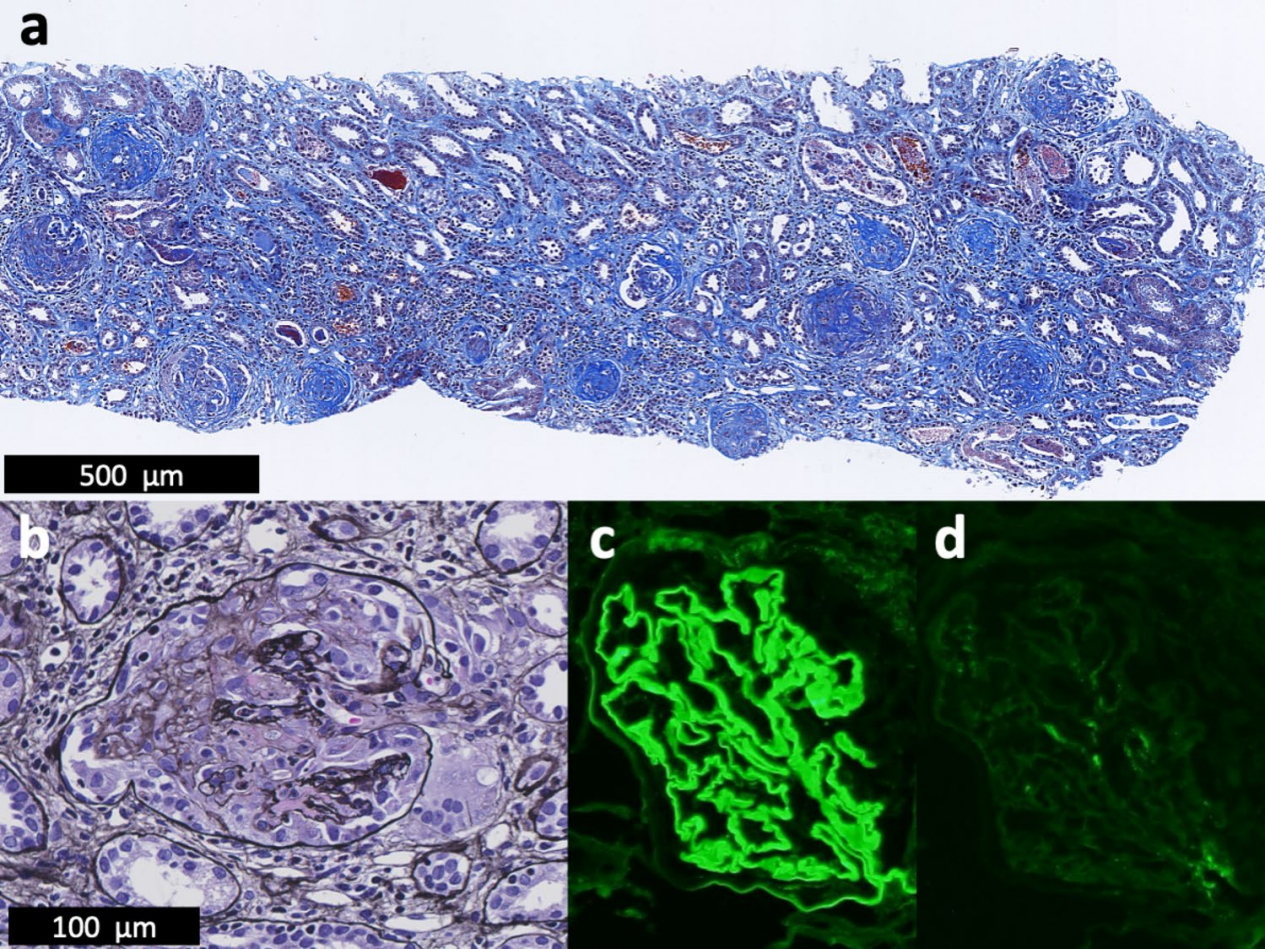
Kidney histopathology findings. **a** Masson’s trichrome staining showing globally sclerotic glomeruli with extensive interstitial fibrosis. **b** A cellular/fibrocellular crescent observed in a residual glomerulus on periodic acid–methenamine silver (PAM) staining. **c**, **d** Immunofluorescence microscopy demonstrating linear IgG deposition along the glomerular capillary walls, with no IgA deposition in the mesangial areas

She was initially treated as IgAVN with prednisolone (60 mg/m^2^/day) and mizoribine (5 mg/kg/day), an immunosuppressive agent that inhibits lymphocyte proliferation and is widely used in Japan [3]. Because kidney biopsy procedures are performed only on specific scheduled days at our institution, the biopsy was carried out at the earliest possible opportunity, 6 days after transfer. Kidney biopsy revealed crescentic glomerulonephritis. Of 52 glomeruli, only 4 were normal, while 28 showed global sclerosis. Fibrous crescents were present in 6 glomeruli, and fibrocellular crescents in 16 (Fig. 1). Immunofluorescence demonstrated linear IgG deposition along the GBM without mesangial IgA deposition. At approximately the same time, the anti-GBM antibody levels returned markedly elevated (≥ 640 IU/mL), confirming the diagnosis of anti-GBM nephritis. No clinical signs of alveolar hemorrhage were observed, with no evidence of hemoptysis and a normal chest X-ray without pulmonary infiltrates.

Treatment was initiated with daily plasma exchange and immunosuppressive therapy consisting of prednisolone (1 mg/kg/day) and cyclophosphamide (2.5 mg/kg/day, with a cumulative dose of 177.5 mg/kg), along with methylprednisolone pulse therapy (30 mg/kg/day, administered 3 days per week for 3 weeks). Anti-GBM antibody levels progressively decreased and became undetectable by day 27 after transfer, allowing discontinuation of plasma exchange. Kidney function temporarily improved; however, despite sustained antibody negativity, kidney function gradually deteriorated, and peritoneal dialysis was initiated 5 months after diagnosis.

## Discussion

This case illustrates the diagnostic challenge of anti-GBM nephritis when urinary abnormalities arise during follow-up of another glomerular disease such as IgAV. Because anti-GBM nephritis progresses rapidly, early recognition is essential.

The KDIGO guidelines emphasize that kidney prognosis in anti-GBM nephritis depends largely on the extent of irreversible glomerular injury at diagnosis, and they recommend against aggressive therapy in patients presenting with dialysis dependence and extensive chronic lesions in the absence of alveolar hemorrhage [2]. However, these recommendations should not be interpreted as absolute contraindications to treatment. Recent literature suggests that even patients with a very low proportion of preserved glomeruli may benefit from individualized aggressive therapy when ongoing immune activity is suspected [4]. In our patient, despite advanced pathological changes at diagnosis, intensive treatment was initiated to suppress antibody production and preserve residual kidney function. Although long-term kidney survival was not achieved, temporary improvement was observed, supporting the concept that therapeutic nihilism should be avoided.

An important consideration is whether the hematuria and proteinuria observed after IgAV onset but before COVID-19 represented early anti-GBM nephritis or IgAVN. It is possible that anti-GBM nephritis existed at an early stage but remained clinically unrecognized. Alternatively, IgAV may have caused glomerular injury and immune activation [5], and subsequent COVID-19 may have further contributed to immune dysregulation, leading to the production of anti-GBM antibodies. Glomerular damage may expose cryptic antigens within the basement membrane, thereby promoting autoantibody formation [6]. However, based on the available clinical data, it is difficult to definitively distinguish between these possibilities. We consider IgAVN more likely, as kidney function remained preserved during this period. Given the aggressive nature of anti-GBM nephritis, a more rapid decline in kidney function would have been expected if the disease had already been present. Although mesangial IgA deposition was absent on biopsy, previous reports indicate that IgA deposits may diminish early in IgAVN [7]. Thus, the clinical course supports de novo development of anti-GBM nephritis later in the disease course.

Emerging epidemiological data suggest an increased incidence of anti-GBM nephritis since the COVID-19 pandemic [8]. Although a causal relationship cannot be established in this case, the temporal association between SARS-CoV-2 infection and rapid kidney deterioration warrants attention. These trends suggest that clinicians may encounter anti-GBM nephritis more frequently in the post-COVID-19 era, including in pediatric populations. Vigilant monitoring and early screening for RPGN are therefore essential when urinary findings or kidney function worsen, even during routine follow-up of pre-existing glomerular diseases.

This case provides an important clinical insight for pediatric nephrologists. Worsening urinary abnormalities or kidney dysfunction during follow-up of IgAV should not be automatically attributed to IgAVN. Even in the presence of a pre-existing glomerular disease, early consideration of RPGN and prompt serological screening, including testing for anti-GBM antibodies, as well as timely kidney biopsy, are essential to avoid diagnostic delay and irreversible kidney injury. Increased awareness of anti-GBM nephritis, particularly in the post-COVID-19 era, may facilitate earlier diagnosis and preserve therapeutic opportunities, even in pediatric patients in whom the disease is considered rare.

## Summary

### What is new?

- Anti-GBM nephritis may develop during follow-up of IgA vasculitis. Early screening for RPGN is essential when urinary findings worsen. Aggressive treatment should be considered even in advanced disease.

## Data Availability

Data sharing is not applicable to this article, as no datasets were generated or analyzed during the current study.
